# Inhibitory control is affected by reward in patients with alcohol use disorder

**DOI:** 10.3389/fpsyt.2025.1496519

**Published:** 2025-06-13

**Authors:** Yalei Li, Xu Cai, Yinzhao Liu, Liping Jia, Guohua Lu

**Affiliations:** ^1^ Department of Public Health, Shandong Second Medical University, Weifang, China; ^2^ Department of Psychosomatic Medicine, Shandong Daizhuang Hospital, Jining, China; ^3^ Department of Biomedical Sciences of Cells and Systems, University Medical Center Groningen, University of Groningen, Groningen, Netherlands; ^4^ Department of Psychology, Shandong Second Medical University, Weifang, China

**Keywords:** alcohol use disorder, reward, punishment, inhibitory control, oddball

## Abstract

**Introduction:**

The present study aimed to investigate the effects of reward and punishment on inhibitory control in the alcohol use disorder (AUD) group and healthy control group.

**Methods:**

Eighteen male patients with AUD and twenty-one age- and education-matched male healthy controls were recruited for the study. Participants engaged in the two-choice oddball paradigm, which included reward, punishment, and neutral conditions. Participants were asked to respond differently to standard and deviant stimuli as accurately and quickly as possible.

**Results:**

For reaction time measures, deviant - standard difference of the healthy control group did not show any difference; however, deviant - standard difference of the AUD group was significantly larger in the reward condition than in the neutral condition. For accuracy measures, deviant - standard difference of the healthy control group did not show any difference; however, deviant - standard difference of the AUD group was significantly larger in the neutral condition than in the reward condition, indicating a greater decline in accuracy for deviant stimuli.

**Conclusion:**

Our findings demonstrated that either reward nor punishment effectively enhanced inhibitory control in AUD patients. Notably, the reward condition was associated with a further decline in inhibitory control. It is advisable to avoid relying solely on reward- or punishment-based behavioral correction strategies, as they might heighten psychological stress and negative emotions, potentially worsening deficits in inhibitory control.

## 1 Introduction

Alcohol use disorder (AUD) is a prevalent psychiatric condition characterized by significant reliance on alcohol, leading to impaired brain function. This disorder commonly leads to various physical ailments, mental disabilities, and cognitive impairments (1–4). With a global incidence of 5.1%, the disease affects a billion people worldwide. In recent years, the burden of the disease has been increasing, making it a significant global public health concern (5). Previous studies showed that, despite the availability of psychological and pharmacological treatments, a substantial proportion of AUD patients failed to achieve sustained improvement. Even after receiving treatment, AUD patients often exhibited high relapse rates and poor prognoses (6, 7). Due to the high relapse rates and poor prognosis characteristic, identifying effective strategies for promoting and maintaining abstinence is of critical importance (8).

AUD was closely associated with widespread cognitive impairments, particularly in executive function. These executive dysfunctions often persisted even after prolonged periods of abstinence. Brion et al. (9) found that individuals with AUD exhibit varying degrees of impairment across the three core components of executive functions, including shifting, updating, and inhibition A review further highlighted the acute and specific detrimental effects of alcohol on executive function, with inhibitory control being especially vulnerable (10). Inhibitory control is a crucial aspect of the execution function, which pertains to a capacity to suppress responses to irrelevant stimuli while engaging in the task of focusing on specific stimuli (11). It also encompassed the ability to effectively restrain preferential responses or interference with information. Notably, impairments or deficits in inhibitory control exacerbated alcohol use and craving (12–15), and was significantly associated with an increased risk of relapse among AUD patients (16).According to the dual-process model, the onset and maintenance of AUD were closely related to the interaction between impulsive and control cognitive systems (17). The impulsive system rapidly evaluated reward-related cues in the environment, such as alcohol-related stimuli, which triggered automatic cravings and impulsive behaviors. In contrast, the control system operated more slowly and rationally, working to inhibit these immediate impulses (18). With repeated exposure to addictive substances, the impulsive system became increasingly dominant, while the control system was progressively weakened. This shift in the balance between the two systems contributed to the development and persistence of addictive behaviors. Furthermore, the incentive sensitization theory posited that repeated exposure to addictive substances induced neural sensitization, rendering individuals increasingly reactive to alcohol and related cues in an automatic and compulsive manner (19, 20).

In addition, several studies demonstrated that reduced inhibitory control was a significant predictor of addictive behaviors and was associated with an increased risk of developing AUD (21). Therefore, enhancing inhibitory control might not only support the maintenance of abstinence and reduce relapse risk among AUD patients, but also serve as a preventive strategy against impulsive drinking behaviors and the onset of AUD. In clinical practice, some studies focused on developing interventions aimed at improving inhibitory control, with the goal of enhancing treatment outcomes. Strengthening inhibitory control could improve cognitive regulation in AUD patients, diminish the dominance of the impulsive system in addictive behaviors and processes, and thus offered new perspectives and directions for clinical intervention.

Integrating motivational factors into research on inhibitory control deficits in AUD held significant theoretical and clinical relevance. Such integration contributed to a deeper understanding of the mechanisms underlying the development and maintenance of alcohol dependence and AUD, and provided valuable guidance for the development of more effective intervention and treatment strategies (22). While increasing attention was directed toward the influence of reward- and punishment-based motivation in shaping inhibitory control in AUD patients, existing findings remained limited and inconclusive.

Motivational factors, particularly monetary rewards, could enhance inhibitory control and improve reaction times. Individuals who showed increased sensitivity to the rewarding effects of alcohol also displayed increased sensitivity to non-drug rewards, specifically stimuli related to monetary reward (23–25). According to the dual competition model proposed by Pessoa, cognitive processing resources were inherently limited and subjected to competition between perceptual and executive demands (26). Both emotional and motivational factors influenced inhibitory control by modulating the allocation of these limited resources. Specifically, motivation could redirect cognitive resources toward reward-related tasks, thereby maximizing potential outcomes. Rossiter and colleagues (27) conducted a comparison of response inhibition over rewarding stimuli between harmful and non-hazardous alcohol users by using a go/no-go paradigm under neutral, reward, and punishment conditions. They found no significant difference between harmful and non-hazardous alcohol users under the neutral condition. During the punishment condition, individuals who engage in harmful alcohol use shown a notably diminished ability to control their impulses in response to rewarding stimuli. However, their performances significantly improved during the delayed reward condition. The results further demonstrated that rewards have the potential to significantly enhance inhibitory control and accuracy of responses in AUD patients (27).

However, certain studies failed to observe any improvement of the inhibitory control ability, which was regulated by the presence reward and punishment, in AUD patients. A fMRI study revealed that the inhibitory control of participants was not influenced by the presence of reward or punishment. Furthermore, no discernible alterations were observed in the brain regions within the insula, dorsal and ventral striatum between groups (28). The researchers posited that the variability in findings could be attributed to the neuroadaptations and modifications in brain circuitry resulting from prolonged and excessive alcohol intake (29). These neuroadaptation alterations might manifest as inhibition for ventral striatum activity (30). Poulton and colleagues (31) used the monetary incentive control task to replicate drinking scenarios in both binge alcohol users and control participants, which pushed individuals to forgo immediate benefits in order to obtain delayed rewards. The finding indicated that individuals who engaged in binge drinking exhibited a notably diminished ability to limit their responses when compared to the control group, regardless of the specific settings involving reward. Hypoactivity in the frontoparietal region had a significant impact on defective reward processing, resulting in worse behavioral performance in both reward and loss contexts in those who engage in binge drinking. Therefore, how reward and punishment affect inhibitory control in AUD patients remains unclear and controversial, warranting further in-depth investigation.

Another important point to note was that previous studies primarily relied on the go/no-go paradigm and the stop-signal task (SST) to assess inhibitory control. However, both approaches were subject to specific methodological limitations. In the go/no-go paradigm, participants were typically instructed to respond as quickly and accurately as possible to go stimuli and to withhold responses to no-go stimuli. The evaluation of inhibitory control was based primarily on the accuracy of no-go trials. Specifically, the assessment of inhibitory control in the go/no-go task was primarily based on the failure to withhold responses in no-go trials, without incorporating reaction time as a comparative measure.This design limited the sensitivity in detecting inhibitory control deficits, often resulting in non-significant differences between go and no-go conditions (32, 33). Moreover, as button-press responses were required only in go trials, accuracy could be easily confounded by behavior-related noise, further undermining the reliability of the assessment (34–36). By contrast, the SST introduced reaction time measures to improve the precision of inhibitory control assessment. In this paradigm, participants were instructed to respond quickly and accurately to go signals. On a subset of trials, a stop signal followed the go stimulus, requiring participants to inhibit an initiated response or suppress an already-activated motor impulse (37, 38). Inhibitory control was quantified by the stop-signal reaction time (SSRT), which reflected the estimated time needed to halt an ongoing response. However, SSRT was not directly observable; instead, it was inferred from the difference between the mean reaction time on go trials and the stop-signal delay (SSD) associated with a 50% probability of successful inhibition. Moreover, the SST primarily assessed inhibitory control in response to external cues, emphasizing reactive, signal-dependent inhibition, where behavior was terminated upon receiving an external stop signal (39). In real-world contexts, however, behavioral inhibition often relied on proactive, self-regulatory processes that were guided by internal goals, rules, or motivational factors such as anticipated reward or punishment. As such, the SST had inherent limitations in capturing spontaneous, self-initiated forms of inhibitory control.

To address these limitations, the two-choice oddball paradigm (TCO) was proposed as an effective tool for assessing inhibitory control (34). In the TCO paradigm, participants were required to make distinct responses to both standard and deviant stimuli, rather than responding solely to go stimuli. This design ensured balanced behavioral responses for both stimuli types, effectively controlling for behavioral preparation and execution-related confounds. Consequently, the TCO paradigm reduced the influence of motor-related factors on behavioral measures and enhances the interpretability and specificity of the results. Inhibitory control was typically assessed by examining the differences in reaction time and accuracy between deviant and standard stimuli.

In the present study, we adopted the two-choice oddball paradigm to elicit inhibitory control processes, aiming to investigate the effects of different motivational factors on inhibitory control in AUD patients. This research sought to provide novel insights for the development of more effective interventions in relapse prevention and rehabilitation programs for AUD patients. We hypothesized that in the neutral condition (without reward or punishment), AUD patients would exhibit impaired inhibitory control compared to healthy controls, primarily reflecting deficits in inhibitory control in reaction time and accuracy. Both reward and punishment conditions would enhance inhibitory control in AUD patients, indicating that motivation could improve inhibitory control.

## 2 Materials and methods

### 2.1 Participants and design

A total of 46 participants were recruited to participate in the present study. Five AUD patients and two healthy controls were excluded from the database due to missing data and mismatching criteria. Due to difficulties in recruiting female participants, the final sample comprised 18 male patients [age (years): *M* = 47.83, *SD* = 4.68, range = 39–55], who were admitted to the Department of AUD at Shandong Daizhuang Hospital between August and November 2022. These participants constituted the AUD group. These participants constituted the AUD group. The inclusion criteria for the AUD group were as follows: met the Diagnostic and Statistical Manual of Mental Disorders 5 (DSM-5) diagnostic criteria for AUD; no history of other substance abuse; receiving non-local medical treatment; patients or family members provided informed consent; an education level above junior high school and available to complete the relevant assessments and tasks; inpatients with a hospitalization period of more than one month who had already passed the acute withdrawal phase; normal hearing and normal or corrected-to-normal vision. The exclusion criteria were: comorbid severe physical and brain diseases; current or past comorbid psychiatric disorders, such as depressive disorders, anxiety disorders, bipolar disorder, or psychotic disorders, with the exception of AUD; family history of epilepsy and seizures; poor compliance, noncooperation, and resistance to the study; having participated in similar experiments or had experience of training on inhibitory control or attentional control.

21 male health participants with matched ages and years of education were recruited in the local community as the healthy control group [age(years): *M*=46.00, *SD*=3.67, range=37-59]. The inclusion criteria of the health control group were: no history of other substance abuse; education level above junior high school and availability to finish the relevant assessments and tasks; consent to participate in the study; a score lower than 8 on the alcohol use disorders identification test (AUDIT; 40, 41); normal hearing and normal or corrected-to-normal vision. The exclusion criteria were: history of neurological disorders or severe physical illness; history of alcohol and drug abuse or dependence; previous involvement in similar experiments or previous experience with inhibitory control or attentional control training; current or past diagnosis of AUD; typically consuming more than 10 standard alcohol units per week (with one unit equivalent to 10 grams of pure ethanol), and more than 3 units per day.

The sociodemographic data, which included ages and years of education, were collected from both groups. The present study was approved by the hospital medical ethics committee. A 2 (group: AUD group, healthy control group) × 3 (trial type: reward, punishment, neutral) × 2 (stimuli type: deviant stimuli, standard stimuli) mixed experimental design was carried out. An *a priori* power analysis using G*Power v3.1.9.7 indicated that a sample size of 28 was required to detect a group × trial type × stimuli type interaction in a repeated-measures ANOVA, assuming a medium effect size (*f* = 0.25), with 0.95 power and *α* = 0.05. This criterion was met in the present study.

### 2.2 Experimental procedure

The experimental program was prepared and run by E-Prime 2.0 using a Lenovo Think Vision desktop computer with a display resolution of 1024 × 768 pixels.Participants were seated directly in front of the computer screen at a viewing distance of 60 cm.The viewing angle was 10°. All participants were right-handed and were instructed to perform all button-press responses using their right hand.

The two-choice oddball paradigm was conducted to investigate the capacity of the participants from both groups. There were two phases to the experiment. The first phase was the practice phase, which included 15 trials, and the second was the experimental phase with 300 trials. These trials were under reward, punishment, and neutral conditions in turn. The practice phase was designed to helping participants understand the trial procedure.

The experimental phase included reward, punishment, and neutral conditions. In the neutral condition, firstly, the center of the screen showed a fixation “+” for 500 ms, followed by a black triangle or a circle for 1500 ms. The circle was the standard stimulus with an 80% probability of presence, and the triangle was the deviant stimulus with a 20% probability of presence. Participants were asked to respond to the figures by pressing the “F” key for a circle and the “J” key for a triangle. If participants responded to the stimuli within the corresponding time, the figure would disappear after pressing the key. If participants did not respond, they would move on to the next trial after 1500 ms. The feedback from the oddball paradigm was shown afterward for 500 ms.

The feedback presented different contents according to the responses of participants and condition requirements. In the reward condition, when responding correctly and fast, participants could obtain more points, including “+1”, “+2”, “+3”, “+4”, and “+5”. In contrast, when responding incorrectly or losing, participants only obtain a “+0” point. In the punishment condition, the faster the participants respond, the fewer points were deducted, including “-0”, “-1”, “-2”, “-3” and “-4”. If participants responded incorrectly or failed to respond, a penalty of -5 points was applied. Finally, participants earned money by accumulating points. The presentation order of each block was balanced between participants, and there was a 5-minute break between blocks.

### 2.3 Measure

The Chinese version of the alcohol use disorders identification test was used for assessing alcohol consumption and use problems and to preliminary screen for the presence of AUD (42). The original English version was recommended by WHO in 1989 and was widely used in clinical evaluation. There were 10 items on this scale. Items 1–8 were scored on a 5-point Likert scale, and items 9–10 on a 3-point scale. The AUDIT score was the cumulative score of all items. A higher score on the scale reflected more severe AUD symptoms. The AUDIT score of more than 8 indicated the presence of alcohol use problems and AUD. The Chinese version of the AUDIT has been widely used, and its reliability and validity have been well established in multiple studies (43, 44). And the Cronbach’s alpha was 0.98 in the present study.

### 2.4 Statistical analysis

Data analyses were conducted using SPSS version 26.0 and R software.The differences in sociodemographic characteristics between the AUD group and the healthy control group were assessed by *χ^2^* for categorical variables and the *t* test for continuous variables. Correlation analyses were also performed to examine the relationships among continuous variables. Behavioral data were analyzed using repeated-measures ANOVA, with group (AUD group vs. healthy control group) as the between-subjects factor and trial type and stimuli type as within-subjects factors. To assess performance consistency and intraindividual variability, we computed the intraindividual coefficient of variation (ICV), calculated as the standard deviation of reaction times divided by the mean reaction time (SDRT/MRT), which controls for individual differences in overall response speed (45).The two-sided *p* value was declared significant if it was less than 0.05. The Greenhouse-Geisser correction was used to compensate for sphericity violations, and *post-hoc* tests were holm-bonferroni test corrected.

## 3 Results

### 3.1 Statistical analysis

There were no significant differences in age or years of education (*Ps* > 0.05). But there was a significant difference between the two groups on AUDIT scores [*t* (38) = -44.78, *P* < 0.001, *d* = 1.81]. Further analysis showed that AUDIT scores of the AUD group were significantly higher than the healthy control group, indicating that the grouping of the present study was valid.

Correlation analyses revealed that AUDIT scores were positively correlated with reaction times for deviant stimuli in the neutral (*r* = 0.53, *P* < 0.001), reward (*r* = 0.54, *P* < 0.001), and punishment conditions (*r* = 0.59, *P* < 0.001), as well as for standard stimuli in the neutral (*r* = 0.49, *P* = 0.002), reward (*r* = 0.37, *P* = 0.02), and punishment conditions (*r* = 0.43, *P* = 0.006). In contrast, AUDIT scores were negatively correlated with accuracy for deviant stimuli in the reward condition (*r* = -0.40, *P* = 0.01), and for standard stimuli in both the reward (*r* = -0.46, *P* = 0.004) and punishment conditions (*r* = -0.35, *P* = 0.03). Additionally, AUDIT scores were positively correlated with reaction time difference (deviant stimuli minus standard stimuli) in the reward condition (*r* = 0.37, *P* = 0.02), and with accuracy difference in the neutral condition(*r* = 0.33, *P* = 0.04).

### 3.2 Reaction time

We first removed error data and extreme data with more than 2.5 standard deviations for both groups. The mean reaction times of each group for the standard and deviant stimuli were shown in **Table 1**. A 2 (group: AUD group vs. healthy control group) × 3 (trial type: reward vs. punishment vs. neutral) × 2 (stimuli type: deviant stimuli vs. standard stimuli) repeated-measures ANOVA results showed a significant group main effect [*F*_(1,37)_ = 15.77, *P* < 0.001, *η^2^* = 0.23], reaction time was significantly longer in the AUD group than in the healthy control group (*P* < 0.05). There was a significant trial type main effect [*F*_(2,74)_ = 4.52, *P* = 0.02, *η^2^* = 0.02], with reaction times significantly longer in the neutral condition than in the punishment condition(*P* < 0.05). And there was a significant stimuli type main effect [*F*_(1,37)_ = 93.29, *P* < 0.001, *η^2^* = 0.25], reaction time was significantly longer in the deviant stimuli than in the standard stimuli. Moreover, there was also a significant trial type × stimuli type interaction effect [*F*_(2,74)_ = 4.71, *P* = 0.01 *η^2^* = 0.003] and a significant group × trial type × stimuli type interaction effect [*F*_(2,74)_ = 4.35, *P* = 0.02, *η^2^* = 0.003]. To further observe the differences in inhibitory control performance between the two groups, we performed the reaction time difference (deviant stimuli minus standard stimuli) and conduct a repeated-measures ANOVA analysis. The results showed that deviant - standard difference of the healthy control group did not show any difference (*Ps >* 0.05). However, deviant - standard difference of the AUD group was significantly larger in the reward condition than in the neutral condition (*P <* 0.05), while no other differences were significant (*Ps* > 0.05).This indicated that reward condition failed to improve inhibitory control in the AUD group.

**Table 1:** The mean reaction time, mean accuracy, mean deviant - standard difference in each condition among AUD group and healthy control group, *M±SD*.

Variables	AUD group			Healthy control group		
	Deviant stimuli	Standard stimuli	Deviant - standard difference	Deviant stimuli	Standard stimuli	Deviant - standard difference
RT Neutral, ms	541.66 ± 19.47	456.41 ± 20.27	85.24 ± 15.18	446.65 ± 18.02	363.00 ± 18.77	83.65 ± 14.05
RT Reward, ms	538.56 ± 23.59	406.55 ± 20.37	132.01 ± 17.38	421.97 ± 21.84	338.37 ± 18.86	83.60 ± 16.09
RT Punishment, ms	529.18 ± 18.19	416.34 ± 20.63	112.84 ± 16.56	430.40 ± 16.84	337.68 ± 19.10	92.36 ± 15.33
ACC Neutral, %	0.84 ± 0.15	0.94 ± 0.17	-0.10 ± 0.13	0.78 ± 0.19	0.99 ± 0.01	-0.22 ± 0.19
ACC Reward, %	0.72 ± 0.19	0.94 ± 0.10	-0.22 ± 0.13	0.84 ± 0.12	0.99 ± 0.01	-0.16 ± 0.12
ACC Punishment, %	0.72 ± 0.21	0.96 ± 0.06	-0.24 ± 0.17	0.81 ± 0.15	0.99 ± 0.04	-0.17 ± 0.15

RT, Reaction time; ACC, Accuracy; M, Mean; SD, Standard deviation. “Deviant – standard difference” refers to the subtraction of the performance metrics (RT or ACC) for standard stimuli from those for deviant stimuli in each condition.

The ICV for all participants were analyzed. And the results showed that the healthy control group exhibited ICV values below 0.2 across all conditions, ranging from 0.16 to 0.18, indicating relatively stable reaction times. However, ICV values across all conditions in the AUD group were consistently above 0.2, ranging from 0.21 to 0.23, suggesting greater variability in reaction times and higher response time instability. These findings might indicate significant deficits in inhibitory control among AUD patients, as reflected by increased intraindividual variability.

### 3.3 Accuracy

The mean accuracy among the two groups was shown in **Table 1**. The results revealed a significant stimuli type main effect [*F*_(1,37)_ = 88.53, *P* < 0.001, *η^2^* = 0.34]. Further analysis showed that the accuracy of deviant stimuli was significantly lower than that of standard stimuli (*P* < 0.001). There was also a significant group × trial type interaction effect [*F*_(2,74)_ = 6.31, *P* = 0.004, *η^2^* = 0.02] and a significant group × trial type × stimuli type interaction effect [*F*_(2,74)_ = 8.21, *P* = 0.002, *η^2^* = 0.03]. Further analysis of three-way interaction effect revealed that the accuracy of deviant stimuli was significantly lower than that of standard stimuli in healthy control groups and across all conditions (*Ps* < 0.05). In the AUD group, the accuracy of deviant stimuli was significantly lower than that of standard stimuli in the reward and punishment conditions (*Ps* < 0.05). To further observe the differences in accuracy between the two groups, we performed the accuracy difference (deviant stimuli minus standard stimuli) and conduct a repeated-measures ANOVA analysis. The results showed that deviant - standard difference of the healthy control group did not show any difference (*Ps* > 0.05). However, deviant - standard difference of the AUD group was significantly larger in the neutral condition than in the reward condition (*P* < 0.05). These indicated that the AUD group showed a greater decline in accuracy for deviant stimuli, and that the reward condition failed to improve their inhibitory control.

Correlation analyses were conducted to explore the speed-accuracy trade-off. The results showed a significant positive correlation between reaction time and accuracy for deviant stimuli in the neutral condition, in both the overall sample (*r* = 0.33, *P* = 0.04) and in the healthy control group (*r* = 0.49, *P* = 0.03). In the AUD group, a significant positive correlation was found between reaction time and accuracy for standard stimuli in the neutral condition (*r* = 0.62, *P* = 0.006). These findings suggest that, healthy control group was able to improve their response accuracy for more difficult deviant stimuli by slowing down their reaction times in the neutral condition, indicative of a flexible cognitive control strategy. In contrast, AUD group could only compensate for accuracy by slowing down their responses during relatively simple and low-demand tasks (i.e., standard stimuli). No significant correlations between reaction time and accuracy were observed in the reward or punishment conditions in either group (*Ps* > 0.05). These results indicated that the impaired inhibitory control observed in the reward conditions was not attributable to a speed-accuracy trade-off.

## 4 Discussion

The present study used a two-choice oddball paradigm to investigate the effects of reward and punishment on inhibitory control in the AUD group compared to the healthy control group. The two-choice oddball paradigm effectively elicited behavioral inhibition in both groups, and the inhibitory control performances of AUD group were worse in reward condition compared to the neutral condition. Therefore, it demonstrated that the inhibitory control of the AUD group was susceptible to reward, potentially leading to inferior behavioral performance.

We found that, regardless of reaction time and accuracy, there were no significant differences between the behavioral performances of two groups in the neutral condition. It demonstrated that inhibitory control was similar in both groups. The dual process theory (24) distinguished between impulsive and control processes, and proposed that the interaction between these cognitive processes was intimately associated with individual addictive behaviors and exerted a significant influence on the addiction cycle. The control system functioned as a regulatory mechanism, akin to a braking system. If the impulsive system consistently became stronger while the control system concurrently weakened, addictive behaviors would persist. Subsequently, the development of addiction or dependence on alcohol in individuals resulted in detrimental effects on brain regions associated with cognitive function, namely the frontal lobe, parietal lobe, limbic system, and cerebellum, with variable degrees of severity. One of the indications of brain injury was executive dysfunction, which included decreased inhibitory control. This illness was characterized by patients experiencing challenges in controlling dominant responses, particularly in relation to the difficulty of inhibiting the immediate pleasure and rewards associated with drinking, as well as the temptation to consume alcohol promptly. These difficulties had been found to significantly impact the likelihood of relapse and recurrence following a period of abstinence. Brion and colleagues (9) utilized a model to investigate the functions of inhibitory control. They conducted a comparison of behavioral performance between the AUD group and normal group. The results revealed that the AUD group exhibited significantly inferior performance on tasks related to inhibition, renewal, and transfer. However, this finding was inconsistent with the present study, which did not reveal differences in inhibitory control in behavioral data between two groups.

The present result was consistent with the study of Lannoy et al. (46). Their study included a go/no-go paradigm wherein alcohol cans and soda cans were utilized as the go stimuli and the no-go stimuli, respectively. The aim of their investigation was to examine and compare the inhibitory control capacities of individuals with alcohol addiction and those who are healthy, particularly in relation to the processing of alcohol-related cues. No significant differences in inhibitory control were observed between the two groups, as evidenced by the accuracy and reaction time results. The researchers suggested that although alcohol addiction leads to varying levels of brain impairment, the brain has the capacity to employ alternate mechanisms to mitigate the detrimental effects induced by alcohol. The compensatory system described facilitated efficient task processing and addressed the difficulties encountered by the brain, specifically in inhibiting dominant responses. As a result, the behavioral data between both groups appear similar.

Another aspect to consider was that the AUD patients who participated in this study were selected from inpatient units. It is crucial to recognize that these patients might have a strong, ongoing desire to leave the hospital, which might have influenced both their conscious and unconscious motivations. The findings of the study were highly regarded, and there was a strong incentive to allocate extra resources to mitigate the harmful consequences of chronic alcohol addiction. Hence, the behavioral data might not have accurately reflected subtle changes in inhibitory control.

Notably, the results of the present study indicated that the reward condition failed to enhance inhibitory control in AUD patients, as evidenced by increased reaction times and a greater decline in accuracy. One possible explanation was that the reward might function as a source of distraction rather than facilitation in certain contexts. Although participants were only eligible for potential rewards during trials involving high-value information, the decline in accuracy during these trials resulted in a net decrease in overall gains. In other words, participants performed better in the neutral condition than in the reward condition, reflecting a paradoxical effect. This pattern of impaired performance might reflect an automatic attentional capture mechanism rather than a deliberate, strategic adjustment of behavior (47, 48). Even when participants were explicitly instructed that reward-related information was not task-relevant and should be ignored, they still struggled to suppress attention toward this high-value information. Once such information was detected, it automatically attracted and occupied cognitive resources, rapidly capturing attention and impairing the timely disengagement from value-related distractors (49). This excessive attentional fixation significantly undermined participants’ focus and responsiveness to task-relevant goals (50). Consequently, in the reward condition, AUD patients experienced greater difficulty disengaging from high-value feedback due to automatic attentional capture, which further disrupted resource allocation and impaired their inhibitory control.

Another possible explanation was that AUD patients showed a reduction in motivation toward non-alcohol rewards, which weakened the facilitative effect of rewards on their inhibitory control. Previous researches showed that AUD patients exhibited significantly lower activation of the reward system when processing non-alcohol reward, such as monetary reward, compared to healthy individuals. As a key brain region involved in reward processing, the ventral striatum showed reduced activation to non-alcoholic reward cues (51–53). Moreover, this phenomenon might be due to neurobiological changes in the brain reward system. Chronic and excessive alcohol consumption led to adaptive remodeling of the mesolimbic-cortical reward circuit, where the density of postsynaptic dopamine D2 receptors was reduced, leading to impaired phasic dopamine signaling and a decrease in overall dopamine release and regulation capacity (54–56). This dysfunction in the dopaminergic system weakened sensitivity to non-alcohol rewards and impaired the reward learning mechanism, meaning that even when non-alcohol external rewards were provided, the motivation system and cognitive resources of AUD patients were not effectively activated, and their inhibitory control was not significantly enhanced. Further researches found that the weakened response of the ventral striatum to reward signals was closely related to alcohol craving, impulsivity, and the severity of AUD (51, 53, 57). Consistent with the results of this study, the correlational analysis also showed that as the severity of AUD increased, reaction times in the reward condition were significantly prolonged, and accuracy further declined, indicating that non-alcohol rewards had a limited effect on enhancing inhibitory control.

Furthermore, another explanation worth considering was the allostatic hypothesis (58, 59), which posited that prolonged consumption of addictive substances triggered a response in the brain counter-reward network, which was a counter-adaptive process, in addition to overstimulating the reward system (60, 61) and contributing to the development of addiction. The activation of a specific region within the brain counter-reward network could potentially impact the inhibitory control of AUD patients, ultimately leading to suboptimal behavioral performance. An fMRI study indicated that individuals with AUD had a decrease in activation of the frontal lobe and insula, as well as a decrease in activation of the ventral striatum, which was associated with the reward system (52, 62–65). Consequently, it was more probable that reward had a detrimental effect on the behavioral performance of inhibitory control rather than enhancing it. The precise mechanisms underlying neuronal and brain networks remained uncertain. The results of the current study bring attention to the potential challenges associated with exclusively providing positive reward, such as immediate financial reward or long-term improvement in overall well-being, as a means to promote abstinence in individuals with AUD. This approach might prove challenging to sustain abstinence and could potentially heighten the likelihood of relapse. This was due to the negative impact on the brain reward system and the activation of counter-reward networks within the brain. It is advisable for future research on clinical alcohol abstinence to take a prudent stance towards the implementation of rewards.

The results of present study showed that the punishment condition did not enhance inhibitory control in the AUD group. One possible reason for this result was that, similar to reward, punishment might also serve as a distraction or interference in certain contexts (66). When individuals detected stimuli or feedback with punishment value, it often triggered an automatic attentional capture response, making it difficult to disengage attention from these irrelevant punishment-related information in a timely manner (49). This continued focus on punishment-related distractors occupied a large portion of limited cognitive resources, leading to a distraction from the goals of task, and making it difficult for individuals to effectively inhibit the interference effects, thereby failing to enhance inhibitory control.

Another possible reason was that AUD patients exhibited lower prefrontal cortex activation and impaired functional connectivity with key brain regions, such as the striatum, which hindered the effectiveness of punishment in enhancing inhibitory control. Punishment situations often triggered heightened stress or anxiety, and previous research showed that both acute and chronic stress could disrupt prefrontal cortex function, leading to a broad range of cognitive impairments (67). Although such stress responses might serve an adaptive function by facilitating rapid reactions in threatening situations, they tended to impair advanced cognitive functions, particularly inhibitory control and flexible decision-making (68–70). These impairments were primarily linked to elevated monoamine neurotransmitters (e.g., norepinephrine and dopamine) and glucocorticoids, which suppressed prefrontal neurons activity and disrupted inhibitory control (69, 70). For example, Tong et al. (28) used a stop-signal paradigm and monetary incentive delay task, and both behavioral data and fMRI data revealed no significant differences between patients and healthy controls in the reward and loss conditions. The frontal regions were inactive, and inhibitory control remained low. Additionally, abnormal functional connectivity between the striatum and prefrontal cortex also made it difficult for AUD patients to improve inhibitory control. Previous study showed that during a monetary incentive delay task, AUD patients exhibited significantly lower ventral striatum activity compared to healthy controls when avoiding losses (65). Park et al. (71) suggested that the functional coupling between the striatum and prefrontal cortex was crucial for adaptive decision-making and learning. In AUD patients, dysfunction in this pathway appeared to hinder the regulatory effect of punishment or negative feedback, thereby limiting improvements in inhibitory control in the punishment conditions.

Based on the present findings, clinical interventions for AUD patients should have adopted a more comprehensive approach. Rather than relying solely on behavior modification techniques based on reward and punishment feedback, which might have increased cognitive load and impaired inhibitory control, treatment would have benefited from strategies that strengthened goal-directed behavior and cognitive regulation. Methods such as motivational interviewing could have helped patients establish and maintain abstinence goals while enhancing self-regulatory capacity. Additionally, incorporating stress-management and coping skills training might have alleviated stress-related dysfunction in the prefrontal cortex, thereby improving executive functioning and inhibitory control. Collectively, these strategies might have provided more effective means of supporting sustained abstinence and reducing relapse risk in individuals with AUD.

There were several limitations in the present study. First, the AUD patient group consisted entirely of males with a wide age range, and the small sample size might have contributed to increased individual variability in the behavioral data, thereby limiting the interpretability and generalizability of the findings. Due to the challenges associated with recruiting inpatient participants, the sample was primarily drawn from male inpatient units, limiting the ability to examine gender-related differences in inhibitory control. Future research could aim to broaden the sampling framework, achieve a more balanced gender distribution, and conduct stratified analyses across different age groups to enhance the representativeness and explanatory power. Second, the stimuli used in this study were relatively simple, which might have resulted in a ceiling effect, particularly among healthy control participants, leading to minimal differences in inhibitory control across experimental conditions. This might have limited the sensitivity to detect subtle variations of inhibitory control in the reward and punishment contexts. Future studies could be encouraged to increase task complexity or diversify stimuli characteristics in order to better capture the dynamic interplay between reward processing and inhibitory control mechanisms in AUD patients. Additionally, the present study employed a purely behavioral paradigm, relying primarily on measures such as reaction time and accuracy. While informative, these indicators were insufficient to elucidate the underlying neural mechanisms involved in reward and punishment processing during inhibitory control. Future studies could benefit from incorporating cognitive neuroscience techniques, such as fMRI and event-related potentials (ERP), to further examine the roles of the prefrontal cortex, striatum, and other relevant neural circuits. Such approaches could have provide a more robust neurobiological foundation for the development of targeted interventions and treatment strategies for AUD patients.

## 5 Conclusion

In conclusion, the present study demonstrated that neither reward nor punishment effectively enhanced inhibitory control in AUD patients. Notably, the reward condition resulted in a further decline in inhibitory control. Clinical intervention strategies for AUD patients should adopt a comprehensive approach that integrates reward and punishment mechanisms and cognitive control training. Sole reliance on punishment-based behavioral correction should be avoided, as it might increase psychological stress and negative affect, potentially exacerbating deficits in inhibitory control.

## Data Availability

The raw data supporting the conclusions of this article will be made available by the authors, without undue reservation.
