# Supplementary material for: Inhibitory control is affected by reward in patients with alcohol use disorder
Source: Front Psychiatry. 2025 Jun 13;16:1496519. doi: 10.3389/fpsyt.2025.1496519 (PMC12203117; doi:10.3389/fpsyt.2025.1496519)
Supplement: Supplementary file 1 [file Table1.docx]

***Supplementary Material***

1. **Supplementary Tables**

Supplementary Table 1. Demographic and AUDIT for alcohol dependence group and healthy control group（*M*±*SD*）

| Variable | Alcohol dependence group | Healthy control group | *χ²/t* | *P* |
| --- | --- | --- | --- | --- |
| Age (years) | 47.83±4.68 | 46.00±3.67 | 11.97 | 0.68 |
| Education (years) | 9.33±1.75 | 9.86±2.15 | 2.34 | 0.31 |
| AUDIT | 28.94±3.00 | 5.05±1.28 | -33.19 | <0.001 |

Supplementary Table 2. Repeated measures ANOVA results for reaction time

| Effect | MSE | *F* | *P* | *η^2^* | post hoc |
| --- | --- | --- | --- | --- | --- |
| group | 31104.54 | 15.77 | <0.001 | 0.23 | RT_AUD group_>RT_HC group_^***^ |
| trial type | 4559.85 | 4.52 | 0.02 | 0.02 | RT _neutral_ >RT _punishment_^*^ |
| stimulus type | 6021.64 | 93.29 | <0.001 | 0.25 | RT _deviant stimulus_>RT _standard stimulus_^***^ |
| group × trial type | 4559.85 | 0.04 | 0.93 | <0.001 | RT_AUD group, neutral_ > RT_HC group, neutral_^**^; RT_AUD group, reward_ > RT_HC group, reward_^*^; RT_AUD group, punishment_ > RT_HC group, punishment_^**^ |
| group × stimulus type | 6021.64 | 1.33 | 0.26 | 0.01 |  |
| trial type × stimulus type | 651.66 | 4.71 | 0.01 | 0.003 | RT _neutral, standard stimulus_ > RT _reward, standard stimulus_^**^; RT _neutral, standard stimulus_ > RT _punishment, standard stimulus_^*^ |
| group × trial type × stimulus type | 651.66 | 4.35 | 0.02 | 0.003 | RT_AUD group, neutral, deviant stimulus_ > RT_HC group, neutral, deviant stimulus_^*^; RT_AUD group, reward, deviant stimulus_ > RT_HC group, reward, deviant stimulus_^*^; RT_AUD group, punishment, deviant stimulus_ > RT_HC group, punishment, deviant stimulus_^*^ |

Note: *^*^P*<0.05, *^**^P*<0.01, *^***^P*<0.001; RT refers to reaction time; HC group refers to the healthy control group; AUD group refers to the alcohol use disorder group.

Supplementary Table 3. Repeated measures ANOVA results for deviant - standard Difference of reaction time

| Effect | MSE | *F* | *P* | *η^2^* | post hoc |
| --- | --- | --- | --- | --- | --- |
| group | 12043.14 | 1.33 | 0.26 | 0.03 |  |
| trial type | 1303.29 | 4.71 | 0.01 | 0.02 | RT Difference _neutral_ <RT Difference _reward_^*^ |
| group × trial type | 1303.29 | 4.35 | 0.02 | 0.02 | RT Difference _AUD group, neutral_ <RT Difference _AUD group,_ _reward_^*^ |

Note: *^*^P*<0.05; RT Difference reflects inhibitory control ability and is calculated as the reaction time to deviant stimuli minus the reaction time to standard stimuli; HC group refers to the healthy control group; AUD group refers to the alcohol use disorder group.

Supplementary Table 4. Repeated measures ANOVA results for accuracy

| Effect | MSE | *F* | *P* | *η^2^* | post hoc |
| --- | --- | --- | --- | --- | --- |
| group | 0.06 | 2.31 | 0.14 | 0.03 |  |
| trial type | 0.01 | 1.19 | 0.31 | 0.004 |  |
| stimulus type | 0.02 | 88.53 | <0.001 | 0.34 | ACC _standard stimulus_>ACC _deviant stimulus_^***^ |
| group × trial type | 0.01 | 6.31 | 0.004 | 0.02 | ACC_AUD group, neutral_ > ACC_AUD group, reward_^*^ |
| group × stimulus type | 0.02 | 0.004 | 0.95 | <0.001 |  |
| trial type × stimulus type | 0.01 | 1.72 | 0.19 | 0.01 |  |
| group × trial type × stimulus type | 0.01 | 8.21 | 0.002 | 0.03 | ACC_HC group, neutral, standard stimulus_ > ACC_HC group, neutral, deviant stimulus_^***^;ACC_HC group, reward, standard stimulus_ > ACC_HC group, reward, deviant stimulus_^***^;ACC_HC group, punishment, standard stimulus_ > ACC_HC group, punishment, deviant stimulus_^***^;ACC_AUD group, reward, standard stimulus_ > ACC_AUD group, reward, deviant stimulus_^***^;ACC_AUD group, punishment, standard stimulus_ > ACC_AUD group, punishment, deviant stimulus_^***^ |

Note: *^*^P*<0.05, *^**^P*<0.01, *^***^P*<0.001; ACC refers to accuracy; HC group refers to the healthy control group; AUD group refers to the alcohol use disorder group.

Supplementary Table 5. Repeated measures ANOVA results for deviant - standard Difference of accuracy

| Effect | MSE | *F* | *P* | *η^2^* | post hoc |
| --- | --- | --- | --- | --- | --- |
| group | 0.04 | 0.004 | 0.95 | <.001 |  |
| trial type | 0.02 | 1.72 | 0.19 | 0.02 |  |
| group × trial type | 0.02 | 8.21 | 0.002 | 0.07 | ACC Difference _AUD group, neutral_ >ACC Difference _AUD group, reward_ ^*^ |

Note: *^*^P*<0.05; ACC Difference is calculated as the accuracy for standard stimuli minus the accuracy for deviant stimuli; HC group refers to the healthy control group; AUD group refers to the alcohol use disorder group.
